# Neurologic Imaging in a Patient with Cirrhosis and Altered Mental Status: To CT or Not to CT

**DOI:** 10.1155/2021/5588208

**Published:** 2021-07-30

**Authors:** Alexander Polyak, Serguei Bannykh, Andrew Klein, Vinay Sundaram

**Affiliations:** ^1^Department of Medicine, Cedars-Sinai Medical Center, Los Angeles, CA, USA; ^2^Department of Pathology and Laboratory Medicine, Cedars-Sinai Medical Center, Los Angeles, CA, USA; ^3^Comprehensive Transplant Center, Cedars-Sinai Medical Center, Los Angeles, CA, USA; ^4^Division of Gastroenterology and Comprehensive Transplant Center, Cedars-Sinai Medical Center, Los Angeles, CA, USA

## Abstract

Hepatic encephalopathy represents a continuum of neuropsychiatric symptoms among patients with end-stage liver disease. When a patient with cirrhosis presents with altered mental status (AMS), routine neurologic imaging is not typically recommended, due to low diagnostic yield. Guidance from the American Association for the Study of Liver Disease states that, on initial presentation, brain imaging is not required unless there are other signs of intracranial pathology, including focal neurologic deficits. We present a case of a 61-year-old female with cirrhosis presenting with AMS without focal deficits, in whom neurological imaging revealed a meningioma and subsequent resection led to symptom improvement.

## 1. Introduction

Hepatic encephalopathy (HE) affects 30–45% of patients with cirrhosis and is a leading cause of morbidity and mortality in this population, as well as healthcare costs and resource utilization [1]. The presentation of HE varies from covert HE, which can include sleep-wake disturbances and shortened attention span, to overt HE, which may lead to confusion, disorientation, and behavioral and personality changes [2]. Though a patient with cirrhosis presenting with altered mental status (AMS) is often presumed to have HE, the differential diagnosis includes etiologies such as cerebrovascular accident, intracerebral hemorrhage (ICH), or a structural brain lesion, all of which can present with similar symptoms to HE and may lead providers to order brain imaging [3].

The current literature suggests imaging of the brain should not be performed in a patient presenting to the hospital with suspected HE, as the diagnostic yield has been shown to be low [4–6]. One study reported no findings of acute intracranial abnormalities on the computed tomography (CT) scan in a cohort of patients with cirrhosis and AMS, but without focal neurologic deficits on physical exam [5]. Another investigation reported that, among 178 brain CT scans performed for 152 HE hospitalizations, only 1% of the CT scans led to a change in diagnosis or management [6]. Therefore, the literature does not support routine use of neurologic imaging as part of the evaluation for altered mental status in the patient with cirrhosis. We present a case of a patient with cirrhosis presenting with altered mental status, where a brain CT scan provided the diagnosis and led to changes in management to successfully treat the patient.

## 2. Case Report

A 61-year-old woman with cirrhosis secondary to nonalcoholic steatohepatitis presented to the emergency room (ER) with increased frequency of intermittent confusion and emotional lability for one month. Prior to presenting to the ER, she reported symptoms of not recognizing her children or her husband, as well as occasionally feeling shaky and frustrated with her confusion. She was started as an outpatient on lactulose 30 mL TID three months prior to hospital presentation, due to presumed hepatic encephalopathy. Despite adherence to lactulose, she continued to have episodes of confusion, anxiety and emotional lability.

**Figure 1:**
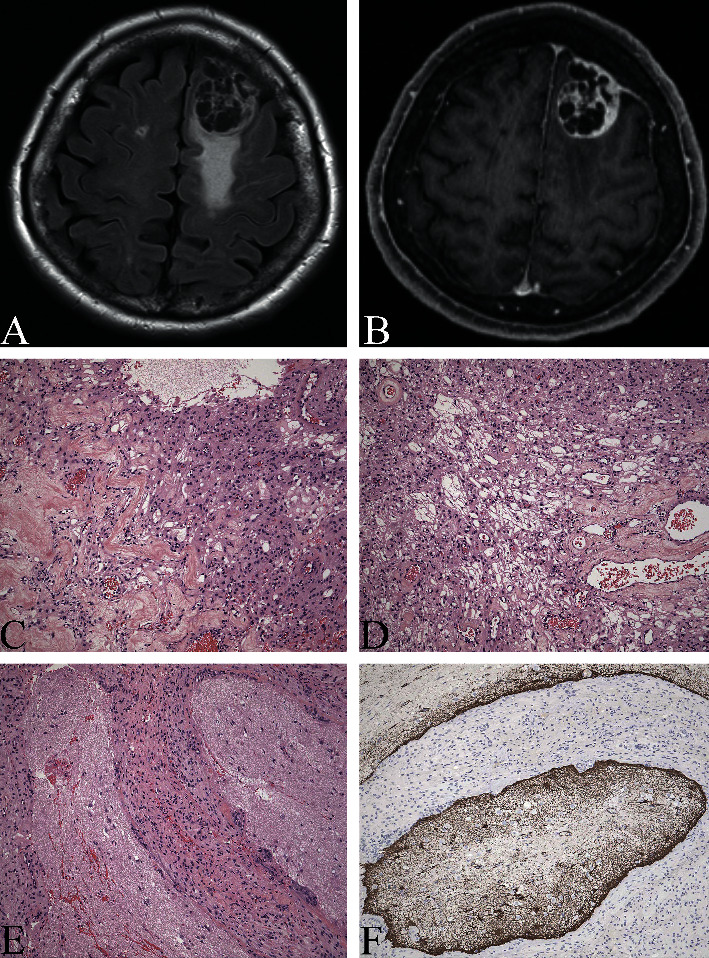
FLAIR (a) and T1 postcontrast (b) MRI images showing dural-based enhancing (b) lesion with associated FLAIR hyperintensity (a). Histology shows angiomatous (c), microcystic (d), and histology with brain invasion (e) as highlighted by GFAP (glial fibrillary acidic protein) immunostain (f). Original magnification (c)–(f): 200x.

On presentation to the ER, she was awake, alert, and oriented to self, place, and date, but was confused as to why she was at the hospital and expressed a tangential thought process. Her complete neurological exam revealed no focal deficits including no cranial nerve deficits, no weakness, no sensory deficits, normal deep tendon reflexes, and no asterixis. Initial laboratory tests demonstrated an ammonia level of 98 *μ*mol/L and a MELD-Na score of 14. She was continued on lactulose, and rifaximin was started. A head CT scan ordered from the ER showed a left frontal convexity extra-axial lesion, measuring 26 mm × 24 mm with underlying parenchymal edema suspicious for a meningioma. Subsequently, an MRI with contrast showed an extra-axial tumor along the left frontal midconvexity measuring 32.3 × 28 mm, associated with FLAIR hyperintensity (Figures 1(a) and 1(b)). Neurosurgery was consulted, and the patient underwent craniotomy and resection of the tumor the next day. Histologic examination revealed an atypical meningioma with predominance of angiomatous and microcystic morphology and foci of brain invasion (Figures 1(c)–1(f)). Her confusion and emotional lability resolved within 48 hours after surgery. She was discharged home and was advised to continue lactulose and rifaximin.

## 3. Discussion

Meningioma is the most commonly diagnosed brain tumor in the United States, with an estimated prevalence of 97.5 per 100,000 people [7]. It has been shown to be associated with changes in cognitive and executive function and can present as new-onset dementia or AMS [8–10]. Medial frontal cortex tumor location has also been associated with increased apathy [10]. The histological findings in our case, atypical meningioma with predominance of angiomatous and microcystic morphology, have also been found to be associated with peritumoral edema, which is known to worsen neurological function [10, 11]. We postulate that the meningioma acted as a precipitant in our patient secondary to associated cerebral edema, leading to worsening of underlying HE. Rapid identification through neurologic imaging was imperative to improving her condition.

Neurologic imaging in a patient with end-stage liver disease presenting with AMS to the emergency room is often performed, but is not usually indicated, particularly in the patient with recurrent presentations for HE [3]. Clinicians may have concern regarding intracranial hemorrhage due to underlying coagulopathy or thrombocytopenia, even though these findings do not translate to an increased risk of bleeding [3, 12]. Studies have shown that the yield of brain imaging in repeat HE admissions is low, specifically when there are no histories of falls or trauma or focal neurologic deficits on exam [5, 13]. Donovan et al. looked at 462 HE admissions and found that the number needed to scan (NNS) for a positive result varied by indication for the scan, showing a low yield if no indication for imaging other than altered mental status: focal neurological deficits (NNS = 9), fall/trauma (NNS = 20), and altered mental status without any other findings (NNS = 293) [14]. A separate study by Rahimi and Rockey evaluated HE patients with no focal neurological deficits and found no difference in mortality if they received brain CT on admission or not [5]. However, these studies have either excluded first-time HE presentations to the hospital or combined both initial and repeat presentations [5, 6, 14]. Although our patient did have symptoms as an outpatient consistent with hepatic encephalopathy, this report represents her first hospital presentation for AMS. Therefore, imaging of the brain was warranted and led to changes in management.

In conclusion, brain imaging is not indicated for patients with recurrent HE admissions without focal deficits on physical exam and without history of trauma or falls, but may have utility in the evaluation of a patient at the time of their initial hospital presentation for AMS.

## Data Availability

No data were used to support this study.
